# Unusual Spreading of Strain Neutral Layer in AZ31 Magnesium Alloy Sheet during Bending

**DOI:** 10.3390/ma15248880

**Published:** 2022-12-12

**Authors:** Chao He, Lintao Liu, Shengwen Bai, Bin Jiang, Hang Teng, Guangsheng Huang, Dingfei Zhang, Fusheng Pan

**Affiliations:** 1 National Engineering Research Center for Magnesium Alloys, College of Materials Science and Engineering, Chongqing University, Chongqing 400044, China; 2 State Key Laboratory of Mechanical Transmissions, Chongqing University, Chongqing 400044, China; 3 Nanjing Yunhai Special Metal Co., Ltd., Nanjing 211212, China

**Keywords:** magnesium, V-bending, strain neutral layer, microstructure

## Abstract

In this work, we reported an unusual phenomenon of strain neutral layer (SNL) spreading in an as-rolled AZ31B magnesium alloy sheet during V-bending. The SNL on the middle symmetrical surface perpendicular to the transverse direction (TD) of the sheet extended to the compression region and was accompanied by a mound-like feature. However, the SNL on the side surface perpendicular to the TD was distributed with a parallel band feature. The underlying mechanism was revealed by the finite element (FE) analysis. The results indicate that the three-dimensional compressive stresses in the compression region of the bending samples were responsible for the above phenomenon. Moreover, the area of the SNL in the middle position gradually decreased as the bending test progressed. The findings in this study provide some new insights into the bending deformation behavior of magnesium alloy.

## 1. Introduction

As a potential lightweight alternative that substitutes for aluminum and high-strength steel alloys, magnesium (Mg) alloys have been greatly developed in recent decades [1,2,3,4]. However, their inherent hexagonal close-packed (HCP) structure would restrict the number of activated slip systems at room temperature (RT) [5,6]. In addition, as one of the most important products of wrought Mg alloys, the sheets prepared by extrusion and rolling always exhibit a strong basal texture [7,8,9]. These factors result in the low formability of Mg alloy sheets at RT, which greatly limits the wide industrial application of Mg alloys [10,11,12]. Therefore, how to improve the formability of magnesium alloys has become a meaningful and urgent topic. During sheet forming, such as deep drawing and hemming, the material at the corner of the part undergoes bending deformation [13,14]. Therefore, bendability is an essential indicator that represents the formability of Mg alloy sheets. The evaluation and improvement of the bendability of Mg alloy sheets has attained adequate attention [15,16,17,18,19,20,21].

During the bending process, there exists a stress gradient through-thickness direction of the bending samples. The tensile stress is distributed in the outer region, while compressive stress is distributed in the inner region of bending samples [17,19,22]. Additionally, the bending samples can be divided into three parts along the thickness direction according to the different strain states, namely compressive strain region (CSR), strain neutral layer (SNL) and tensile strain region (TSR) [17,23,24]. A lot of efforts have been made to investigate the evolution of SNL during the bending of magnesium alloy. For example, Li et al. [16] demonstrated that the SNL tended to first shift toward the outer tensile region, and then shift toward the inner compressive region during the three-point bending of Mg alloy sheets, which is mainly attributed to the asymmetric response of the Mg alloy in tension and compression. Wang et al. [25] investigated the evolution of springback and neutral for AZ31 magnesium alloy by V-bending tests. The results indicate that the SNL shifted to the tension zone of the sample. The offset of SNL decreased with increasing the bending temperature because of the weakening of asymmetry between the tensile and compressive regions. Huang et al. [26] also found that the SNL of the AZ31 magnesium alloy sheet shifted toward the tension region during the V-bending at the temperature of 150 °C. Bai et al. [27] reported that the greater the shift of the neutral layer during the V-bending of the AZ31/Mg-Gd laminated composite sheet, the higher the strain on the outer layer, which makes the sample more prone to fracture. Most of the current studies focus on the shifting of SNL along the thickness direction during the bending. However, the importance of the morphology of SNL is ignored.

The present study observed an unusual phenomenon about the SNL spreading toward the CSR during the V-bending test of AZ31B Mg alloy. Optical microscopy (OM) and finite element (FE) analysis were utilized to investigate the morphology and stress state of the bending samples. It was found that the three-dimensional compress stresses were responsible for the SNL expansion. The underlying mechanisms are discussed based on the FE simulation results.

## 2. Experiments and Simulations

The material used in this study was a hot-rolled AZ31B sheet with a thickness of 3 mm. Table 1 and Table 2 present the chemical compositions and tensile properties along the rolling direction (RD) of the sheet, respectively. Bending samples were machined into strips with the dimension of 50 mm × 12 mm × 3 mm from the as-rolled sheets along the RD. The V-bending test was operated on a universal testing machine (Instron 5985, Boston, MA, USA) at a speed of 1.5 mm/min. The distance between the two supports of the bending tooling was 32 mm. The die angle was 60°, and the radius of the punch was 2 mm. The texture of the as-rolled sheet was examined by the Electron Back-Scattered Diffraction (EBSD, NordlysMax2, Oxford, UK) technique. The microstructures of bending samples were characterized by the optical microscope (OM, ZEISS Axiovert 40 MAT, Oberkochen, Germany). The normal direction (ND)–rolling direction (RD) surfaces of the samples for EBSD and OM measurement were mechanically ground and polished. Thereafter, the OM sample was etched with a solution composed of 1.5 g picric acid, 5 mL acetic acid and 25 mL ethyl alcohol. The EBSD sample was electrochemically polished with the electrolyte AC2 at a voltage of 20 V and a temperature of −25 °C for 90 s.

**Figure 1:**
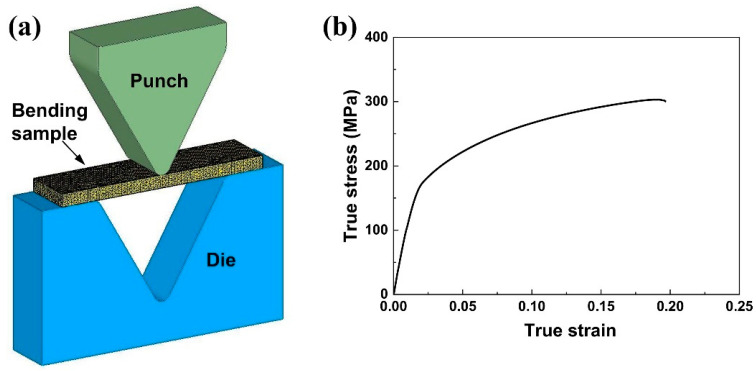
FE model of the V-bending (**a**) and stress–strain data used in the FE simulation (**b**).

FE simulations were performed by the commercial software Deform 3D to analyze the distributions of stress and strain during the V-bending (Figure 1a). In the FE model, the bending sample was regarded as a rigid–plastic body, while both the punch and die were set to be rigid bodies. The bending sample was meshed into tetrahedral elements, and the number of elements was 50,000. The Coulomb friction model was employed at the interface between the sample and tooling, and the friction coefficient was set to 0.3 [28]. The stress–strain data obtained through the tensile test along the RD were input into the model for calculation (Figure 1b).

## 3. Results and Discussion

### 3.1. Bending Behavior of the As-Rolled AZ31 Sheet

**Figure 2:**
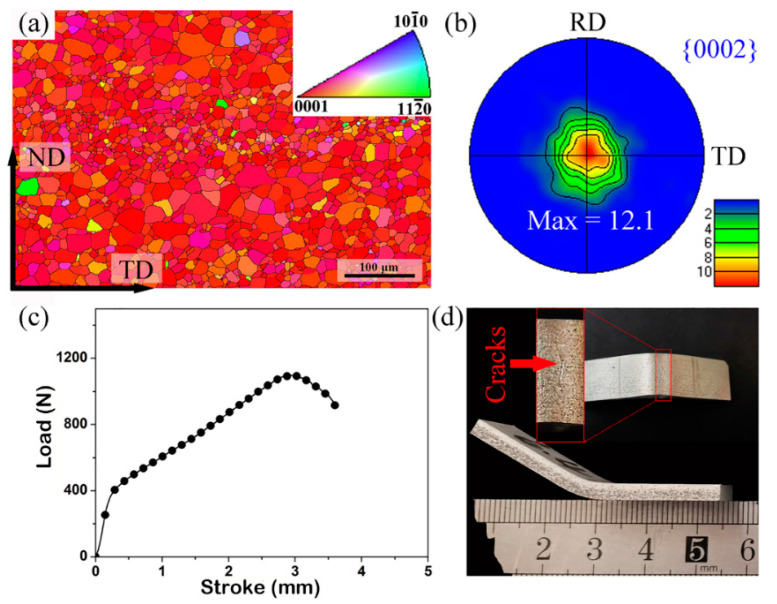
Results of EBSD measurement and bending experiment: (**a**) IPF coloring map, (**b**) (0002) pole figure of the as-rolled AZ31 alloys sheet, (**c**) bending load-stroke curve and (**d**) images of the bent sample.

The initial microstructures of the as-rolled AZ31 sheet are shown in Figure 2a. The completed recrystallized grains are observed, and the average grain size is ~12 μm. In the (0002) pole figure, the *c*-axis of numerous grains are parallel to the ND of the sheet, and the maximum pole intensity is about 12.1 M.U.D (Figure 2b). The typical basal texture is usually formed during the rolling of the Mg alloy [29,30]. Tam et al. [31], for example, also found a strong basal texture with the *c*-axis of most grains parallel to the ND in the rolled AZ31 sheet. The bending load–stroke curve and the corresponding image of the bent sample are shown in Figure 2c,d, respectively. The bending load increases sharply in the initial stage, corresponding to the elastic deformation of the Mg alloy. With further increase in the stroke, the load rises slowly and reaches the peak value due to the work hardening during the plastic deformation. Thereafter, the load is gradually reduced until the test is stopped when the fracture occurs. The maximum stroke before cracking arrived at 3.0 ± 0.2 mm, and the final bending angle is 136 ± 3°. Furthermore, the cracks are located at the middle position of the outer tensile region, as shown in Figure 2d.

### 3.2. Microstructure Evolution during Bending

**Figure 3:**
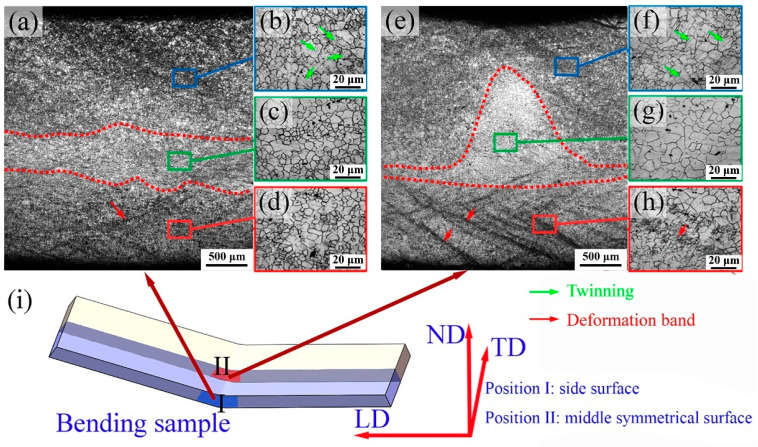
Optical micrographs (**a**–**d**) of the side surface (position I in Figure 3i) and (**e**–**h**) middle symmetrical surface (position II in Figure 3i) perpendicular to the transverse direction (TD) of the sample when the bending test interrupts at the punch stroke of 1.5 mm and (**i**) schematic diagram of the bending sample.

Figure 3 shows the microstructures when the bending test is interrupted at the punch stroke of 1.5 mm. The OM images of the side surface (position I in Figure 3i) of the sample are presented in Figure 3a–d. Figure 3e–h reveals the OM images on the middle symmetrical surface (position II in Figure 3i) perpendicular to the transverse direction (TD). In the TSR, a large number of deformation bands (marked with red arrows) is observed (Figure 3e). Twinning morphologies (marked with green arrows) appear frequently in the CSR (Figure 3b). Furthermore, the SNL without deformation bands or twinning morphologies is located between the TSR and CSR (see Figure 3c). For the as-rolled Mg alloys with strong basal texture, it is widely accepted that the slip dominates the tensile strain, and the {10−12} extension twinning dominates the compressive strain during the bending process. The reason for this phenomenon is that tensile stress in the TSR is perpendicular to the c-axes, which is favorable for the activation of basal and prismatic <a> slips. The {10−12} extension twinning is more likely to be activated under the compression stress state in the CSR [19]. Bai et al. [27] found that extension twinning was activated in the CRS during the bending of the AZ31/Mg–Gd-laminated composite sheet. In the research of Tang et al. [18], a lot of twinning bands were observed in the CSR during the four-point bending of the extruded AZ31 plate, while few twinning bands were formed in the TSR. The phenomena observed in the current study are consistent with previous studies.

Comparing the microstructures at position I and position II, the number of deform bands in the TSR of position II is significantly more than that of position I, which means that the tensile strain at position II is more severe than that at position I under the same punch stroke. The formation of deform bands during the plastic forming of the as-rolled Mg alloy sheets has been reported in many studies. The possible formation mechanisms of these deform bands have been proposed to be related to twinning-induced shear banding (TISB) [32,33] and weak texture-induced shear banding (WTISB) [34,35,36]. The boundaries of SNL are highlighted by the red dotted lines, as shown in Figure 3a,e. The SNL morphologies in these two positions are markedly different. In position I, the boundaries of SNL in Figure 3a are nearly parallel with each other, which is in the region between the CSR and the TSR. This phenomenon is consistent with the results reported by Lee et al. [13], Singh et al. [37] and Jin et al. [23]. For example, Lee et al. [13] observed the side surface of the bent sample using an optical microscope and also found that the boundaries of SNL are nearly parallel with each other. The SNL boundary extends to the compressed region in position II, as shown in Figure 3e. Compared with the position I, the SNL in position II is mound-like, which has never been reported before.

**Figure 4:**
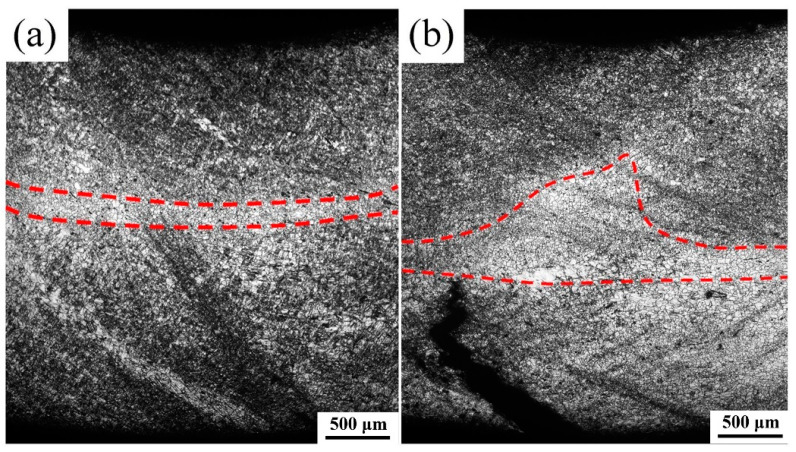
Optical micrographs (**a**) of the side surface (position I in Figure 3i) and (**b**) middle symmetrical surface (position II in Figure 3i) perpendicular to the transverse direction (TD) of the sample when the bending test interrupts at the punch stroke of 3.0 mm.

Figure 4 shows the microstructures when the bending test is interrupted at the punch stroke of 3.0 mm, in which the macrocracks are observed. More deform bands are formed in the TSR as the punch stroke increases from 1.5 mm to 3 mm, no matter whether in position I or position II. This is because the strain in the TSR increases as the bending proceeds. The macrocracks are formed and extended in position II. However, there is no sign of cracks in the side surface (position I) of the bending samples, which is consistent with the images of the bent sample shown in Figure 2d. According to Figure 3 and Figure 4, the tensile strain in the TSR of position II is larger than that of position I during the whole bending test. For the distribution of SNL, a similar phenomenon that parallels the band-like SNL in position I and the mound-like SNL in position II is observed. However, the area of mound-like SNL in position II is decreased with the punch stroke increased from 1.5 mm to 3.0 mm, as shown in Figure 4b.

### 3.3. Simulated Results

**Figure 5:**
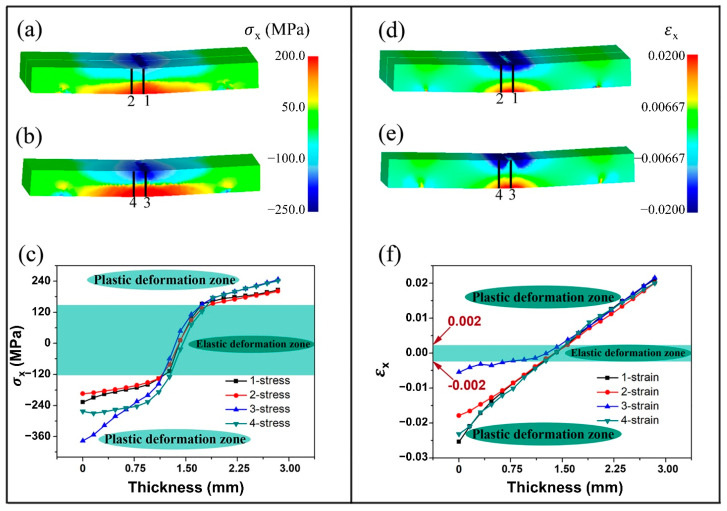
Simulated stress component $\sigma_{x}$ (**a**,**b**) and strain component $\varepsilon_{x}$ (**d**,**e**) in the side position (**a**,**d**) and middle position (**b**,**e**); distributions of stress (**c**) and strain (**f**) along the paths 1, 2, 3 and 4.

To analyze the difference in the SNL between positions I and II, the stress component ($\sigma_{x}$) and strain component ($\varepsilon_{x}$) in the length direction of the bending sample are extracted from the FE simulated results, as shown in Figure 5. Four paths along the thickness of the bending AZ31B sample are defined to better understand the stress distribution during the bending test. Paths 1 and 3 are located at the middle position in positions I and II of the bending samples, respectively. Paths 2 and 4 are situated at the site away from the middle position, about 3 mm from paths 1 and 3, respectively. Figure 5a–c shows the distribution maps and the corresponding detailed curves of $\sigma_{x}$ along the four paths. All the stress curves along the four paths show an ‘S’ shape. This type of stress distribution has been reported by Ren et al. [24]. The higher slope interval that is highlighted by the green shaded area corresponds to the SNL, and the low slope intervals represent the TSR and CSR, as shown in Figure 5c. It is revealed that the stress distribution curves of positions I and II are similar in the TSR. In the CSR, the stress values in position I (paths 1 and 2) are significantly lower than those in position II (paths 3 and 4). This means that the stress distribution along the transverse direction (TD) is not uniform during the bending process. The stress in the middle position is higher than that in the side position, which is consistent with the results observed in Figure 3 and Figure 4. Figure 5d,e show the simulated strain when the punch stroke is 1.5 mm. It is revealed that the boundaries of SNL are nearly parallel with each other in the side position (position I) and arched upward in the middle position (position II), which is consistent with the results of OM observation (Figure 3). Figure 5f shows the strain distribution curves at the two positions along the four paths. Here, we define the elastic strain as the strain with the range of −0.002 ~ 0.002, such that the SNL can be marked by the green shaded area in Figure 5f. In CSR, the strain along path 3 is significantly lower than those along other paths. This means that the SNL extends to the CSR in position II. The simulation results (Figure 3) are highly consistent with the OM observed results.

**Figure 6:**
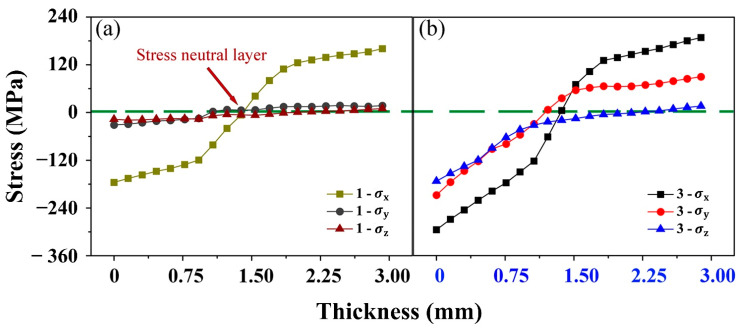
Distributions of $\sigma_{x}$ in the LD, TD and ND along path 1 (**a**) and path 3 (**b**).

The analytical results indicate that the SNL spreading phenomenon of AZ31B magnesium alloy is closely related to the distribution of compression stress during bending. The material is in a relatively stable state under triaxial compression or tension stresses [13,37]. The same idea is used to understand the spreading phenomenon of the SNL in this study. Figure 6 presents the stress components in the length direction (LD), transverse direction (TD) and normal direction (ND) of the bending sample along paths 1 and 3, denoted as $\sigma_{x}$, $\sigma_{y}$ and $\sigma_{z}$, respectively. Along path 1, $\sigma_{x}$ is significantly greater than $\sigma_{y}$ and $\sigma_{z}$. Consequently, the stress component in LD is responsible for the deformation behavior in the position I, and the boundaries of SNL are parallel with each other. For path 3, the difference among the stress components in LD, TD and ND is smaller than that along path 1. In the CSR of position I (corresponding to path 1), the material undergoes a stress state close to the uniaxial compression. The stress state in the CSR of position II is triaxial compression (Figure 6b). In position II, the highest compressive stress components in LD, TD and ND are 298, 199 and 175 MPa, respectively. In the grains under the triaxial compressive stress state, the strain coordination modes, such as basal <a> slip and {10−12} extension twinning, are hard to be activated. The area of the SNL extends to the CSR and forms a mound-like elastic strain region on the middle symmetrical surface perpendicular to TD.

It is defined that the region with a stress value of 0 MPa at the junction of the tensile stress region and compressive stress region of the bending sample is the stress neutral layer. The elastic strain region is defined as the strain neutral layer. However, many reports only considered the stress component in the LD [37,38,39,40,41] and ignored the stress components in the TD and ND. The stress state in the bending sample is much more complex than that of uniaxial tension or compression. Only the three-dimensional stress state is considered, rather than the uniaxial stress separately; the phenomenon of strain SNL spreading can be explained legitimately. The results are important for better understanding the bending behavior of Mg alloy sheets.

## 4. Conclusions

In this work, an unusual phenomenon of SNL spreading is revealed during the V-bending test. The SNL on the middle symmetrical surface perpendicular to TD extends to the compression region with a mound-like boundary. The SNL in the side position is distributed with a parallel band feature. This difference in SNL distribution is mainly attributed to the difference in three-dimensional stress distributions between the side position and the middle position of the bending sample. The three-dimensional compressive stresses in the compressed region are responsible for the SNL spreading phenomenon. An improved FE model, in which the effects of anisotropy and tension–compression asymmetry of the material are taken into consideration, should be constructed in future studies to obtain more insights into the evolution of SNL during the bending of magnesium alloys.

## Figures and Tables

**Table 1:** Chemical compositions of the AZ31B Mg alloy.

Elements	Al	Zn	Mn	Mg
Content (wt.%)	2.87	0.87	0.21	Bal.

**Table 2:** Tensile properties along the RD of the AZ31B Mg alloy sheet.

Yield Strength (MPa)	Ultimate Tensile Strength (MPa)	Elongation (%)
153	303	19.7

## Data Availability

Not applicable.
